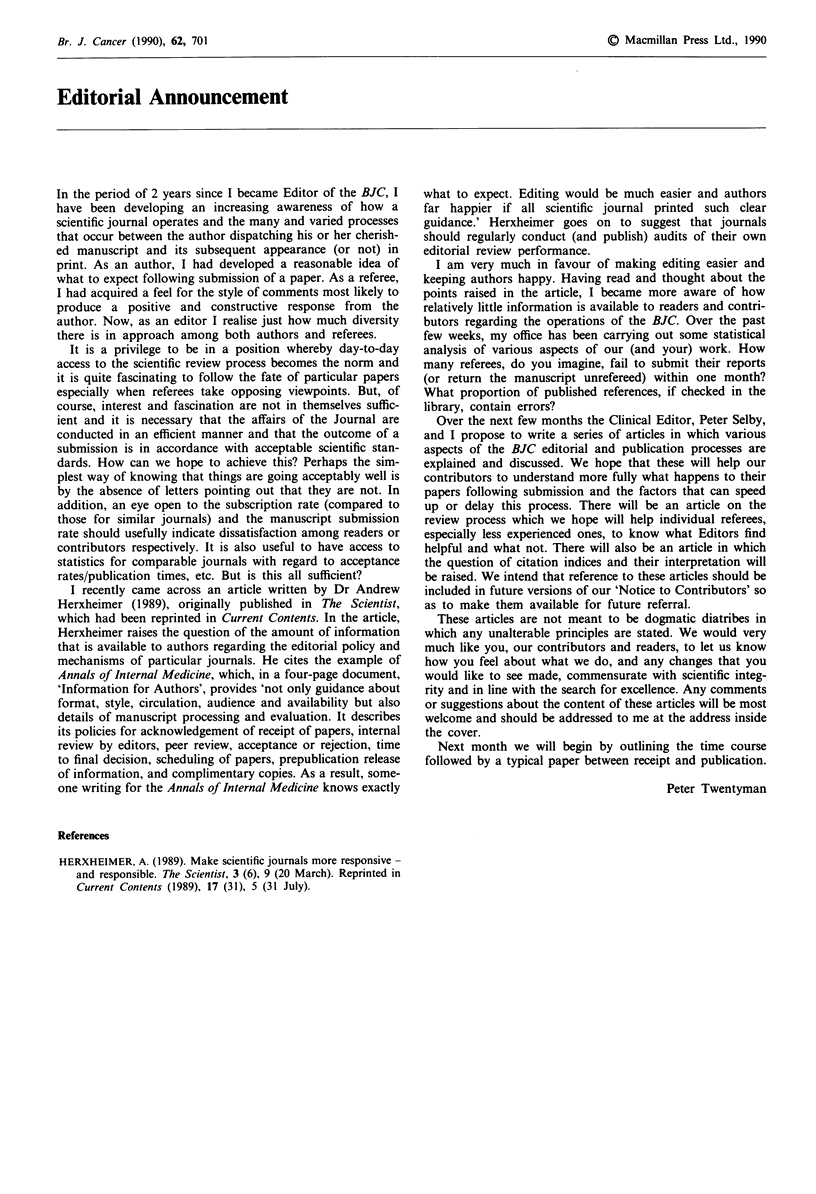# Editorial announcement

**Published:** 1990-11

**Authors:** Peter Twentyman


 © Macmillan Press Ltd., 1990

### Editorial Announcement

In the period of 2 years since I became Editor of the *BJC*, I have been developing an increasing awareness of how a scientific journal operates and the many and varied processes that occur between the author dispatching his or her cherished manuscript and its subsequent appearance (or not) in print. As an author, I had developed a reasonable idea of what to expect following submission of a paper. As a referee, I had acquired a feel for the style of comments most likely to produce a positive and constructive response from the author. Now, as an editor I realise just how much diversity there is in approach among both authors and referees.

It is a privilege to be in a position whereby day-to-day access to the scientific review process becomes the norm and it is quite fascinating to follow the fate of particular papers especially when referees take opposing viewpoints. But, of course, interest and fascination are not in themselves sufficient and it is necessary that the affairs of the Journal are conducted in an efficient manner and that the outcome of a submission is in accordance with acceptable scientific standards. How can we hope to achieve this? Perhaps the simplest way of knowing that things are going acceptably well is by the absence of letters pointing out that they are not. In addition, an eye open to the subscription rate (compared to those for similar journals) and the manuscript submission rate should usefully indicate dissatisfaction among readers or contributors respectively. It is also useful to have access to statistics for comparable journals with regard to acceptance rates/publication times, etc. But is this all sufficient?

I recently came across an article written by Dr Andrew Herxheimer (1989), originally published in *The Scientist*, which had been reprinted in *Current Contents*. In the article, Herxheimer raises the question of the amount of information that is available to authors regarding the editorial policy and mechanisms of particular journals. He cites the example of *Annals of Internal Medicine*, which, in a four-page document, 'Information for Authors', provides 'not only guidance about format, style, circulation, audience and availability but also details of manuscript processing and evaluation. It describes its policies for acknowledgement of receipt of papers, internal review by editors, peer review, acceptance or rejection, time to final decision, scheduling of papers, prepublication release of information, and complimentary copies. As a result, someone writing for the *Annals of Internal Medicine* knows exactly what to expect. Editing would be much easier and authors far happier if all scientific journal printed such clear guidance.' Herxheimer goes on to suggest that journals should regularly conduct (and publish) audits of their own editorial review performance.

I am very much in favour of making editing easier and keeping authors happy. Having read and thought about the points raised in the article, I became more aware of how relatively little information is available to readers and contributors regarding the operations of the *BJC*. Over the past few weeks, my office has been carrying out some statistical analysis of various aspects of our (and your) work. How many referees, do you imagine, fail to submit their reports (or return the manuscript unrefereed) within one month? What proportion of published references, if checked in the library, contain errors?

Over the next few months the Clinical Editor, Peter Selby, and I propose to write a series of articles in which various aspects of the *BJC* editorial and publication processes are explained and discussed. We hope that these will help our contributors to understand more fully what happens to their papers following submission and the factors that can speed up or delay this process. There will be an article on the review process which we hope will help individual referees, especially less experienced ones, to know what Editors find helpful and what not. There will also be an article in which the question of citation indices and their interpretation will be raised. We intend that reference to these articles should be included in future versions of our 'Notice to Contributors' so as to make them available for future referral.

These articles are not meant to be dogmatic diatribes in which any unalterable principles are stated. We would very much like you, our contributors and readers, to let us know how you feel about what we do, and any changes that you would like to see made, commensurate with scientific integrity and in line with the search for excellence. Any comments or suggestions about the content of these articles will be most welcome and should be addressed to me at the address inside the cover.

Next month we will begin by outlining the time course followed by a typical paper between receipt and publication.

Peter Twentyman

### References

HERXHEIMER, A. (1989). Make scientific journals more responsive – and responsible. *The Scientist*, **3** (6), 9 (20 March). Reprinted in *Current Contents* (1989), **17** (31), 5 (31 July).